# Changes in age composition and epidemiology of candidaemia, Iceland, 2012 to 2023

**DOI:** 10.2807/1560-7917.ES.2026.31.40.2600120

**Published:** 2026-10-08

**Authors:** Lena Ros Asmundsdottir, Johanna Gudrun Bergthorsdottir, Andri Oddur Steinarsson, Magnus Gottfredsson

**Affiliations:** 1 Faculty of Medicine, School of Health Sciences, University of Iceland, Reykjavik, Iceland; 2 Department of Clinical Microbiology, Landspitali - University Hospital, Reykjavik, Iceland; 3 Department of Infectious Diseases, Landspitali - University Hospital, Reykjavik, Iceland; 4 Department of Science, Landspitali - University Hospital, Reykjavik, Iceland

**Keywords:** Candidaemia, *Candida*, bloodstream infection, epidemiology, age-standardised incidence, antifungal resistance

## Abstract

**BACKGROUND:**

Candidaemia places a substantial burden on healthcare systems. Long-term, population-based studies of candidaemia rates with appropriate adjustments for changes in age composition are lacking.

**AIM:**

We aimed to determine trends in candidaemia incidence rates, species distribution, antifungal susceptibility, and concurrent antifungal use in Iceland between 2012 and 2023, as well as long-term (2000–2023) trends in age-standardised incidence rates (ASRs).

**METHODS:**

We analysed patient data with blood cultures positive for *Candida* species, or yeasts previously assigned to the genus *Candida*. We calculated ASRs, standardised to the 2023 Icelandic population, and performed susceptibility testing on all available isolates.

**RESULTS:**

Overall, we identified 207 candidaemia episodes in 193 patients. The average ASR was 5.2 cases per 100,000 population. The ASR increased significantly from 2012 to 2023 (incidence rate ratio (IRR) = 1.07; p = 0.001), mainly driven by increased rates among males (IRR = 1.10; p = 0.001). From 2000 to 2023, the ASR remained stable (average 5.8/100,000; IRR = 1.00). The ASR was higher among males, both during 2012–2023 (p = 0.014) and long-term (2000–2023, p = 0.001). Age-specific incidence rate was highest among > 80-year-old males (44.0/100,000). *Candida albicans* was the most frequent species (41.0%), followed by *Nakaseomyces glabratus* (27.2%) and *Candida tropicalis* (11.1%). Fluconazole susceptibility remained high (*C. albicans* 99%, *C. tropicalis* 75%, *Candida parapsilosis* 100%), and echinocandin resistance was rare (1.9%).

**CONCLUSION:**

Despite an apparent increase from 2012 to 2023, candidaemia incidence in Iceland has remained stable in a long-term (24-year) perspective, when appropriate adjustments are made for the ageing population. Concurrently, the epidemiology has shifted towards non-*albicans Candida* infections.

**Key public health message**

**What did you want to address in this study and why?**

Candidaemia is a life-threatening invasive fungal infection that disproportionally affects older patients and those with comorbidities. These population groups are expanding in many countries. Therefore, we adjusted the data for changes in age composition when calculating the occurrence of candidaemia in Iceland from a medium-term (2012–2023) and longer-term (2000–2023) perspective.

**What have we learnt from this study?**

In Iceland, candidaemia increased significantly from 2012 to 2023. However, in a 24-year perspective and when adjusted for population size by age group, the incidence remained stable. The infection was more common among males, and the difference was most pronounced in the oldest age group (> 80-year-olds). There was an increase in cases caused by *Candida* species that are often less susceptible to commonly used antifungal drugs.

**What are the implications of your findings for public health?**

In population-based long-term studies, it is important to make appropriate adjustments for the ageing population to get a correct picture of real trends. The increased number of cases caused by other species than the common *Candida albicans,* and the recent emergence of multidrug-resistant infections is of concern and shows how important it is to continue monitoring these infections and to ensure the appropriate use of antifungals.

## Introduction

Fungal bloodstream infections caused by *Candida* species or yeasts previously assigned to the genus *Candida*, hereafter candidaemia, are a serious health concern in vulnerable patients and place a substantial burden on healthcare systems [1,2]. It is estimated that globally more than 600,000 cases of *Candida* bloodstream infections occur each year, with 165,000 attributable deaths [3]. Variations between and within countries can, however, be large. A meta-analysis of population-based studies of candidaemia in Europe from 2000 to 2019 showed an overall pooled incidence rate of 3.9 per 100,000 inhabitants per year, but varied substantially by patient group, region and over time [4]. In the United States (US), incidence rates have generally been higher. A population-based surveillance for candidaemia in 10 US states from 2017 to 2021 reported an overall incidence rate of 7.4 cases per 100,000 population, which varied by age, sex and ethnicity [5]. Compared with previous surveillance data in the US during the period 2012 to 2016, the rate had not changed substantially, but the all-cause in-hospital mortality had increased [6]. High incidence of candidaemia and different risk factors for the infection have been reported among critically ill COVID-19 patients [7].

The management of candidaemia has been challenged in recent years by shifts in causative species and emerging antifungal resistance. The proportion of infections caused by *Candida albicans* has progressively decreased, and *Nakaseomyces glabratus* (formerly *Candida glabrata*) and *Candida parapsilosis* infections have concomitantly increased [8,9]. In addition, fluconazole-resistant *C. parapsilosis* strains and multidrug-resistant *Candidozyma* (also known as *Candida*) *auris* causing nosocomial outbreaks are associated with adverse patient outcomes and pose substantial challenges for infection control and treatment among hospitalised patients [10,11].

Previous nationwide studies of candidaemia in Iceland showed that after a rapid increase in incidence in the 1980s and 1990s, possibly driven in part by improved detection, the incidence continued to rise, albeit more slowly, during the ensuing 12 years, from 2000 to 2011 [12,13]. There was, however, a notable decrease in incidence after 2009, coinciding with a substantial reduction in the use of blood cultures during economic hardship in the country [13]. The current work aimed to study subsequent trends in incidence rates, species distribution and antifungal susceptibility of candidaemia in Iceland, and the concurrent use of systemic antifungals, in a nationwide setting from 2012 to 2023, a period which also includes the COVID-19 pandemic. An additional aim was to determine long-term trends in the overall, sex- and age-specific incidence rates of candidaemia in the country by reanalysis of data from 2000 to 2011 [13].

## Methods

### Setting, blood culture methods and case definitions

At the beginning of 2012 Iceland had a population of 313,182, and 383,726 (22.5% increase) at the end of 2023 (https://statice.is). From 2012 to 2023, the proportion of inhabitants ≤ 20 years decreased from 29.7% to 26.0%, whereas the proportions aged 21–60 years and > 60 years increased from 53.2% to 54.1% and from 17.0% to 19.9%, respectively. 

Through a nationwide search in microbiology databases, we identified retrospectively all patients in Iceland with blood cultures positive for *Candida* spp. or yeasts previously assigned to the genus *Candida* (hereafter referred to as *Candida*-like), from 1 January 2012 through 31 December 2023. Blood cultures from all patients in the country were processed at the time of collection at two clinical microbiology sites: Landspitali University Hospital (national reference laboratory) and Akureyri municipal hospital. Blood cultures at both sites were performed using the BacT/ALERT (bioMérieux, France) blood culture system. 

An episode of candidaemia was defined as at least one blood culture positive for *Candida* species or yeasts previously assigned to the genus *Candida*. Episodes were considered separate if they occurred at least 30 days apart or were caused by different species [12-14]. Infections were classified as nosocomial or healthcare-associated according to previously published criteria [15].

### Species identification and antifungal susceptibility testing

Yeasts were identified to species level by MALDI-TOF MS (Bruker Nordic AB, Sweden). Antifungal susceptibility testing was performed at the national reference laboratory, where we determined minimum inhibitory concentration (MIC) values with the Sensititre YeastOne microbroth dilution method according to the manufacturer’s instructions (Thermo Fisher Scientific, US). The colourimetric MIC endpoints were read visually using a reading mirror. Because Sensititre YeastOne is based on the Clinical and Laboratory Standards Institute (CLSI) M27 broth microdilution reference method, susceptibility results were categorised according to CLSI MIC breakpoints, where available [16]. In the absence of established CLSI resistance breakpoints, we used species-specific CLSI epidemiological cut-off values (ECVs) to distinguish wild-type (WT) isolates from non-WT isolates that may have acquired mechanisms of resistance [17]. *Pichia kudriavzevii* (formerly *Candida krusei*) was considered intrinsically resistant to fluconazole. Quality control was performed using *C. parapsilosis* ATCC 22019 and *C. krusei* ATCC 6258 strains, and results were within established ranges (Supplementary Table S1) [16].

### Antifungal use

We obtained sales figures from pharmaceutical wholesalers for all antifungal agents for systemic use (anatomical therapeutic chemical (ATC) code J02) in Iceland from the Icelandic Medicines Agency (https://www.ima.is). The numbers should reflect the overall systemic (oral and intravenous) antifungal use in the country, both in hospitals and primary healthcare settings. By using the ATC/DDD system (https://atcddd.fhi.no), sales data was expressed as defined daily doses (DDD) per 1,000 inhabitants per year.

### Statistical analysis

Annual, national demographic data, including sex and age distribution, was obtained from the Icelandic National Registry (https://statice.is/statistics/population/inhabitants/overview). We used the mid-year population (average of the population at the beginning and end of the year) to calculate the population-based incidence rate per 100,000 inhabitants per year (crude and age-standardised incidence rate (ASR)), age-specific incidence rates, and the blood culture rate, both for the current study period (2012–2023) and the preceding 12 years (2000–2011), based on previously published data [13]. We calculated ASRs following the method described by Boyle and Parkin, using the age distribution of the 2023 Icelandic population as the standard population [18]. Briefly, the age-specific incidence rates for each year were weighted according to the 2023 age distribution, thereby adjusting for changes in population age structure over time. Incidence rates of candidaemia per 10,000 patient days and 1,000 admissions were calculated based on data from annual reports of the specialty hospitals in the country. We used a Poisson regression model to examine trends in incidence rates. In cases where diagnostic testing indicated that the models were inadequate, negative binomial regression was used. We compared ASRs for males and females and for different study periods (2000–2011 vs 2012–2023) by calculating the standardised rate ratio (SRR), defined as the ratio of the two ASRs [18]. Proportions were compared by using the Pearson chi-square test and Fisher’s exact test, as appropriate, and chi-square test for linear trend was used to evaluate changes in proportions and rates between groups. We used Pearson’s correlation to examine the linear relationship between MICs for different antifungals. Statistical analyses were performed with IBM SPSS Statistics software (version 31.0.0.0; IBM Corporation, US), Stata (version 17.0; StataCorp LLC, US) and the OpenEpi online epidemiologic statistics software. All p values were based on two-tailed tests and level of significance was set at 0.05.

## Results

### Epidemiology from 2012 to 2023 and a perspective from 2000 to 2011

From 2012 to 2023, 207 episodes of candidaemia were identified in 193 patients in Iceland. In total, 181 patients (93.8%) had a single episode of infection, 11 patients had one recurrence, and one patient had three recurrences. Overall, 185 patients (95.9%) were adults (≥ 18 years). The mean and median age of patients were 60 years (range: 0–96) and 64 years (interquartile range: 49–76), respectively. A majority of patients were male (59.1%; p = 0.012). Overall, 195 episodes (94.2%) were diagnosed at the specialty hospitals in the country. Most cases were classified as nosocomial (146/207; 70.5%) or healthcare-associated (27/207; 13.0%) according to the definitions of Friedman et al. [15]. Across all episodes, patients were most commonly located in medical wards (87/207; 42.0%), intensive care units (49/207; 23.7%) and emergency departments (40/207; 19.3%) when the index blood culture was drawn.

During the 12-year study period, the average crude population-based incidence rate of candidaemia was 5.0 per 100,000 population and the average ASR was 5.2 per 100,000 population (Figure 1A). The ASR increased significantly over the 12 years, with an annual percentage change of 7% (incidence rate ratio (IRR) = 1.07; 95% CI: 1.03–1.12; p = 0.001). The incidence rate per 1,000 admissions and 10,000 patient days at the two specialty hospitals in the country was 0.50 and 0.66, respectively, and both rates increased significantly from 2012 to 2023 (IRR = 1.09; 95% CI: 1.04–1.13; p < 0.001 and 1.09; 95% CI: 1.05–1.14; p < 0.001, respectively). The annual blood culture rate (blood cultures/100,000 population) in the country increased significantly throughout the study period (IRR = 1.06; 95% CI: 1.05–1.06; p < 0.001) (Figure 1A). At the same time the proportion of blood cultures positive for *Candida* spp. increased, from 0.20% in the period 2012 to 2015 to 0.35% in the period 2016 to 2019 and 0.41% in the period 2020 to 2023 (p < 0.001).

**Figure 1:**
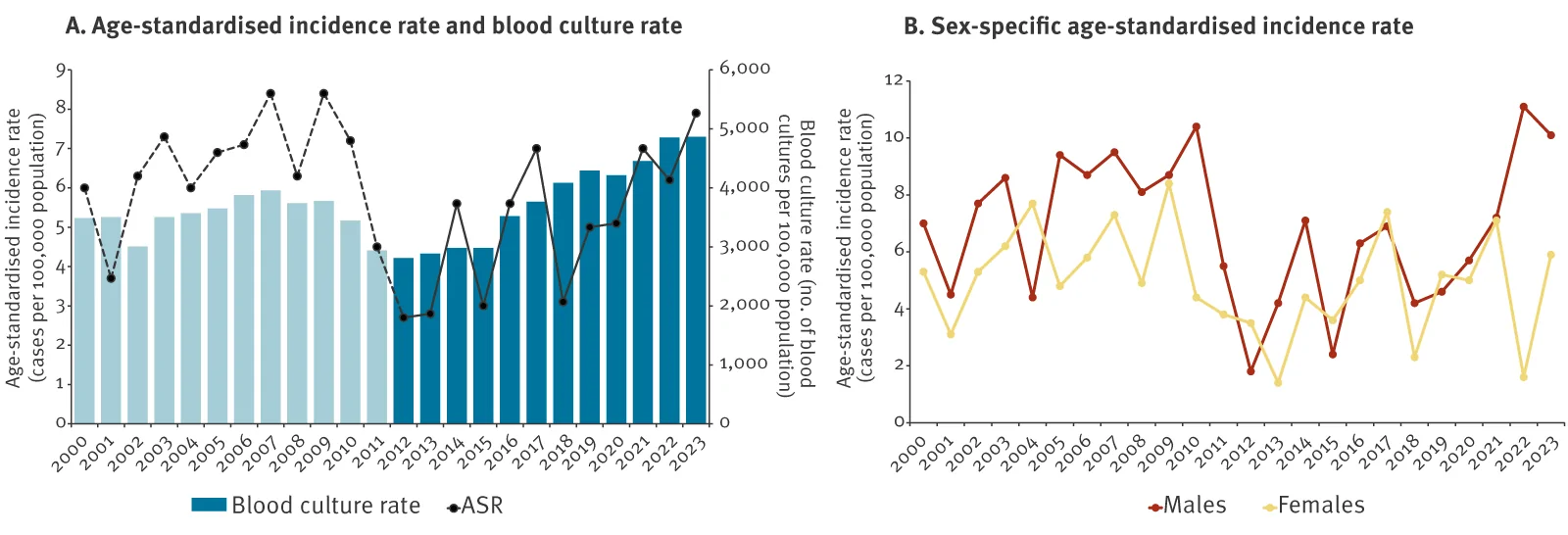
Incidence of candidaemia, Iceland, 2000–2023 (n = 416 episodes)

ASR: Age-standardised incidence rate.

The reference population is the Icelandic population in 2023. For 2000–2011, the incidence rate and blood culture rate in panel A are shown as a dashed line and lighter coloured columns, respectively.

To determine long-term trends in candidaemia incidence, we calculated ASRs for the preceding 12-year period, 2000 to 2011, based on previously published data, standardised to the 2023 Icelandic population [13] (Figure 1A). The ASR from 2012 to 2023 was significantly lower than that from 2000 to 2011 (5.2 vs 6.6 cases per 100,000 population; SRR = 0.79; 95% CI: 0.65–0.96; p = 0.016). However, a separate analysis of annual ASRs across the entire 24-year period showed no significant overall temporal trend from 2000 to 2023 (IRR = 1.00; 95% CI: 0.98–1.01; p = 0.57), with an average ASR of 5.8 per 100,000.

The ASRs were significantly higher among males than females, both during the current 12-year study period (6.2/100,000 vs 4.4/100,000; SRR = 1.41; 95% CI: 1.07–1.86; p = 0.014) and during the extended period from 2000 to 2023 (6.9/100,000 vs 5.0/100,000; SRR = 1.38; 95% CI: 1.14–1.68; p = 0.001) (Figure 1B). For men, the rate increased significantly from 2012 to 2023 (IRR = 1.10; 95% CI: 1.04–1.16; p = 0.001) but was stable in a 24-year perspective (IRR = 1.00, 95% CI: 0.98–1.02; p = 0.98). For women, the ASR remained stable during both time periods.

The highest age-specific incidence rates from 2012 to 2023 were seen in the oldest age groups (13.3/100,000 and 23.6/100,000 for 61–80-year-olds and > 80-year-olds, respectively) (Figure 2). A comparison of incidence rates for all age groups by three quadrennia showed that the rate remained stable, except for > 80-year-olds, where the rate increased significantly from 14.4 per 100,000 in the period 2012 to 2015 to 18.3 per 100,000 in the period 2016 to 2019 and 37.0 per 100,000 in the period 2020 to 2023 (p = 0.028). There was male predominance in most age groups, with the largest age-specific difference observed in the oldest age group (> 80-year-olds; male rate, 44.0/100,000, female rate, 9.1/100,000; p < 0.001). Analysis of age-specific incidence rates in a 24-year perspective (2000–2023), revealed significant but opposite changes in trend in the youngest and oldest age groups, i.e. a decrease among < 1-year-olds (IRR = 0.90; 95% CI: 0.83–0.98; p = 0.020) and an increase among > 80-year-olds (IRR = 1.06; 95% CI: 1.01–1.11; p = 0.018).

**Figure 2:**
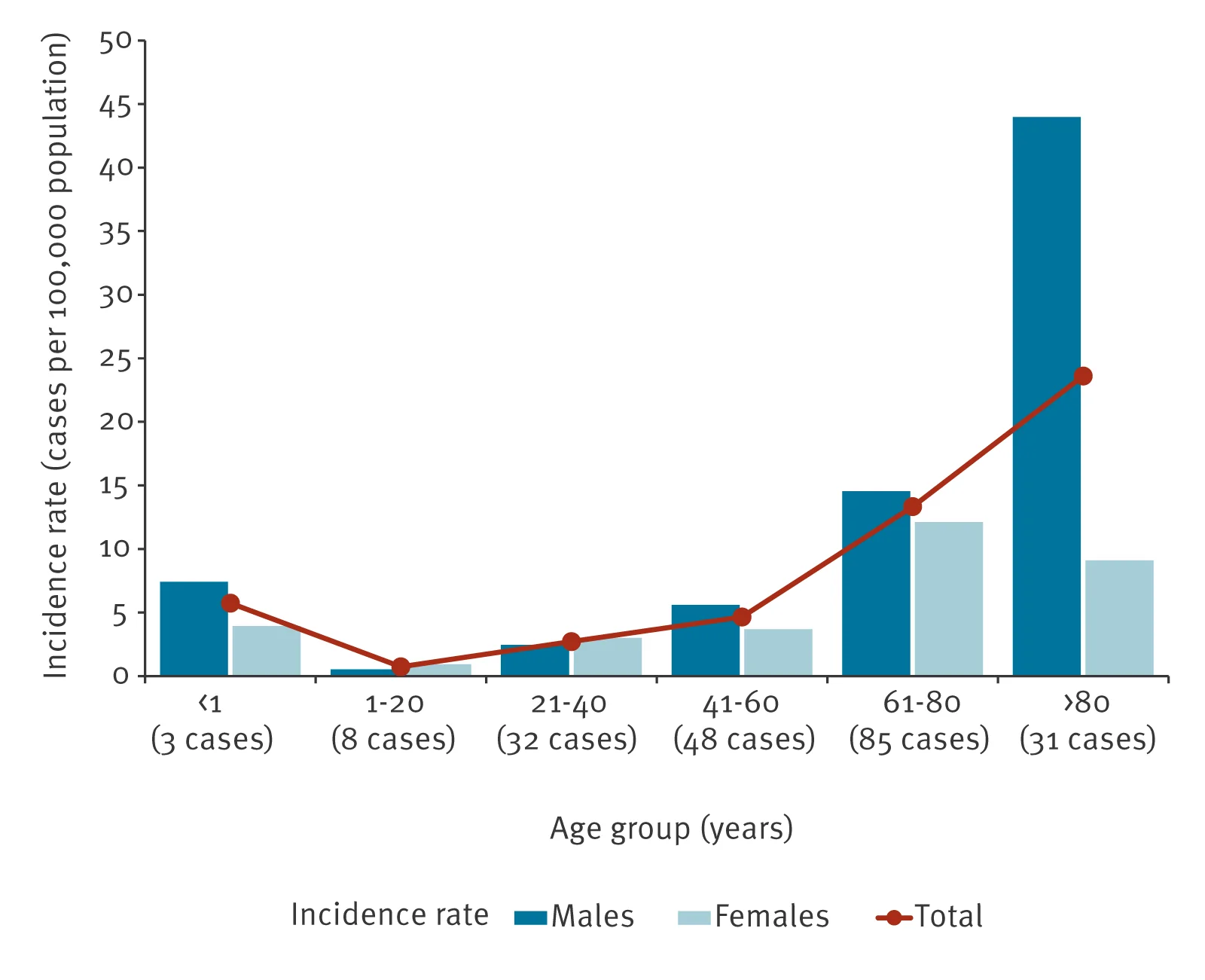
Age- and sex-specific incidence rate of candidaemia, Iceland, 2012–2023 (n = 207 episodes)

### *Candida* and *Candida*-like species

From 2012 to 2023, *C. albicans* was the most frequently isolated species (89/217 isolates; 41.0%), followed by *N. glabratus* (n = 59; 27.2%), *C. tropicalis* (n = 24; 11.1%), *Candida dubliniensis* (n = 20; 9.2%) and *C. parapsilosis* (n = 14; 6.5%) (Table 1). More detailed species distribution is shown in Supplementary Table S2. No *C. auris* was detected. Dual-species infections were identified in 10 of 207 episodes (4.8%) and involved 9.2% (20/217) of the isolates. Overall, the species distribution remained stable throughout the 12-year period. The proportion of species other than *C. albicans* was, however, significantly higher from 2012 to 2023 compared with the preceding 12 years 2000 to 2011 (59.0% vs 44.1%; p = 0.002), mainly due to an increase in the proportion of *N. glabratus* (27.2% vs 16.2%; p = 0.006) [13].

**Table 1:** Species distribution of *Candida* and *Candida*-like bloodstream isolates, Iceland, 2012–2023 (n = 217 isolates)

**Species**	**2012–2015** **(n = 44)**		**2016–2019** **(n = 71)**		**2020–2023** **(n = 102)**		**Total** **(n = 217)**		**p value** **(time trend)**
	n	%	n	%	n	%	n	%	
*Candida albicans*	19	43	33	46	37	36	89	41.0	0.30
*Nakaseomyces glabratus*	12	27	14	20	33	32	59	27.2	0.30
*Candida tropicalis*	6	14	10	14	8	8	24	11.1	0.22
*Candida dubliniensis*	3	7	7	10	10	10	20	9.2	0.62
*Candida parapsilosis*	2	5	4	6	8	8	14	6.5	0.42
Other *Candida/Candida*-like species ^a^	2	5	3	4	6	6	11	5.1	0.67

^a^ Other *Candida*/*Candida*-like species include indicated number of isolates, as follows: *Clavispora lusitaniae* (*Candida lusitaniae*) n = 6; *Meyerozyma guilliermondii* (*Candida guilliermondii*) n = 3; *Pichia kudriavzevii* (*Candida krusei*) n = 1; *Kluyveromyces marxianus* (*Candida kefyr*) n = 1.

Because of rounding, percentages may not total 100 in all instances.

Species distribution of bloodstream isolates according to age group is shown in Table 2. *Candida albicans* was the only species cultured from infants < 1-year-old. For other age groups, there was a trend towards higher proportion of *C. albicans* with increasing age (p = 0.083), whereas the proportion of *N. glabratus* infections was similar between groups. Overall, the species distribution did not vary significantly by sex (data not shown).

**Table 2:** Species distribution of *Candida* and *Candida*-like bloodstream isolates by patient age group, Iceland, 2012–2023 (n = 217)

**Species**	**Age group (years)**											
	< 1 (n = 3)		1–20 (n = 9)		21–40 (n = 34)		41–60 (n = 52)		61–80 (n = 88)		> 80 (n = 31)	
	n	%	n	%	n	%	n	%	n	%	n	%
*Candida albicans*	3	100	3	33	10	29	18	35	36	41	19	61
*Nakaseomyces glabratus*	0	0	3	33	12	35	12	23	24	27	8	26
*Candida tropicalis*	0	0	0	0	7	21	10	19	6	7	1	3
*Candida dubliniensis*	0	0	0	0	2	6	6	12	10	11	2	6
*Candida parapsilosis*	0	0	1	11	1	3	5	10	7	8	0	0
Other *Candida/Candida*-like species ^a^	0	0	2	22	2	6	1	2	5	6	1	3

^a^ Other *Candida*/*Candida*-like speciesinclude indicated number of isolates, as follows: *Clavispora lusitaniae* (*Candida lusitaniae*) n = 6; *Meyerozyma guilliermondii* (*Candida guilliermondii*) n = 3; *Pichia kudriavzevii* (*Candida krusei*) n = 1; *Kluyveromyces marxianus* (*Candida kefyr*) n = 1.

Because of rounding, the total percentage for individual age brackets is not 100 in all instances.

### Antifungal susceptibility

Antifungal susceptibility was determined for 214 (98.6%) of 217 isolates. The results for fluconazole, voriconazole and echinocandins are summarised in Table 3. Results for other antifungals as well as detailed MIC distributions for all antifungals tested are provided in Supplementary Table S3.

**Table 3:** Antifungal susceptibility profiles of *Candida* and *Candida*-like bloodstream isolates for fluconazole, voriconazole and echinocandins, Iceland, 2012–2023 (n = 214)^a^

Species	Fluconazole						Voriconazole						Anidulafungin						Micafungin					
	S or WT	% S or WT	SDD	% SDD	R or NWT	% R or NWT	S or WT	% S or WT	I	% I	R or NWT	% R or NWT	S or WT	% S or WT	I	% I	R or NWT	% R or NWT	S or WT	% S or WT	I	% I	R or NWT	% R or NWT
*Candida albicans* (n = 87)	86	99	0	0	1	1	86	99	0	0	1	1	87	100	0	0	0	0	87	100	0	0	0	0
*Nakaseomyces glabratus* (n = 59)	NA	NA	48	81	11	19	17	29	NA	NA	42	71	57	97	0	0	2	3	58	98	0	0	1	2
*Candida tropicalis* (n = 24)	18	75	2	8	4	17	17	71	5	21	2	8	24	100	0	0	0	0	24	100	0	0	0	0
*Candida dubliniensis* (n = 19)	18	95	NA	NA	1	5	NA	NA	NA	NA	NA	NA	18	95	NA	NA	1	5	18	95	NA	NA	1	5
*Candida parapsilosis* (n = 14)	14	100	0	0	0	0	14	100	0	0	0	0	14	100	0	0	0	0	14	100	0	0	0	0
Other *Candida*/*Candida*-like species (n = 11)^b^	8	73	NA	NA	3^c^	27	NA	NA	NA	NA	NA	NA	10	91	0	0	1^d^	9	10	91	0	0	1^d^	9

CBP: CLSI clinical breakpoints; CLSI: Clinical and Laboratory Standards Institute; ECV: CLSI epidemiological cutoff values; I: intermediate; NA: not applicable; R: resistant; S: susceptible; SDD: susceptible-dose dependent; WT: wild-type; NWT: non-wild-type.

^a^ Classification according to CBP [16]. In the absence of established CBP, we used ECV to distinguish between WT and NWT isolates [17].

^b^ Other *Candida*/*Candida*-like species include indicated number of isolates, as follows: *Clavispora lusitaniae* (*Candida lusitaniae*) n = 6; *Meyerozyma guilliermondii* (*Candida guilliermondii*) n = 3; *Pichia kudriavzevii* (*Candida krusei*) n = 1; *Kluyveromyces marxianus* (*Candida kefyr*) n = 1.

^c^ Includes single NWT *C. lusitaniae* and *M. guilliermondii* isolates, as well as a single *P. kudriavzevii* isolate.

^d^ A single NWT *C. lusitaniae* isolate.

NA indicates that the corresponding category was not defined by the applicable CBP or ECV classification.

#### Azoles

Overall, 144 (67.3%) isolates were susceptible or, for *Candida* and *Candida*-like species without breakpoints, wild-type (WT) for fluconazole. For *C. albicans*, a single isolate was both fluconazole- and voriconazole-resistant and had high MICs to other azoles. Other *C. albicans* isolates were highly susceptible to fluconazole and the distribution of fluconazole MICs did not vary significantly between the study halves (data not shown). Among *C. tropicalis*, four isolates were fluconazole-resistant, two of them also voriconazole-resistant. All *C. parapsilosis* isolates were fluconazole-susceptible. For *N. glabratus*, 11/59 isolates were fluconazole-resistant. The proportion remained stable during the current study period but was significantly higher than in the period 2000 to 2011 (1/36; p = 0.03). All fluconazole-resistant *N. glabratus* isolates were non-WT for voriconazole (MIC > 0.25 mg/L). Significant correlation was found between fluconazole and voriconazole MICs for all species tested (data not shown).

#### Echinocandins

Overall, 98.1% (210/214) of isolates were susceptible (for species without breakpoints, WT) to anidulafungin and micafungin, including all *C. albicans*, *C. tropicalis* and *C. parapsilosis* isolates. Two *N. glabratus* isolates were anidulafungin-resistant. Both were also fluconazole-resistant and non-WT for other azoles, and were thereby multidrug-resistant. Single *Clavispora lusitaniae* (formerly *Candida lusitaniae*) and *C. dubliniensis* isolates were non-WT for echinocandins, and the *C. lusitaniae* isolate was also non-WT for fluconazole. All infections caused by echinocandin-resistant and/or non-WT isolates were nosocomial, and the three multidrug-resistant isolates were cultured in the last 4 years of the study period.

#### Amphotericin B

The CLSI has not established resistance breakpoints for amphotericin B, but all isolates tested were WT for this drug, with MICs at or below the CLSI ECV. The amphotericin B MIC distributions are appended in Supplementary Table S3.

### Antifungal use

Figure 3 displays sales data for systemic antifungal compounds in Iceland from 2012 to 2023. Overall, the use increased by 118%, from 147 DDD per 1,000 inhabitants in 2012 to 320 DDD per 1,000 inhabitants in 2023. The greatest relative increase was seen for echinocandins, from 0.24 to 2.71 DDD per 1,000 inhabitants (1,030%). Micafungin was the main echinocandin used. Anidulafungin was first imported in Iceland in 2016 and accounted for 37% of echinocandin use in the country from 2016 to 2023, reaching 59% in 2022.

**Figure 3:**
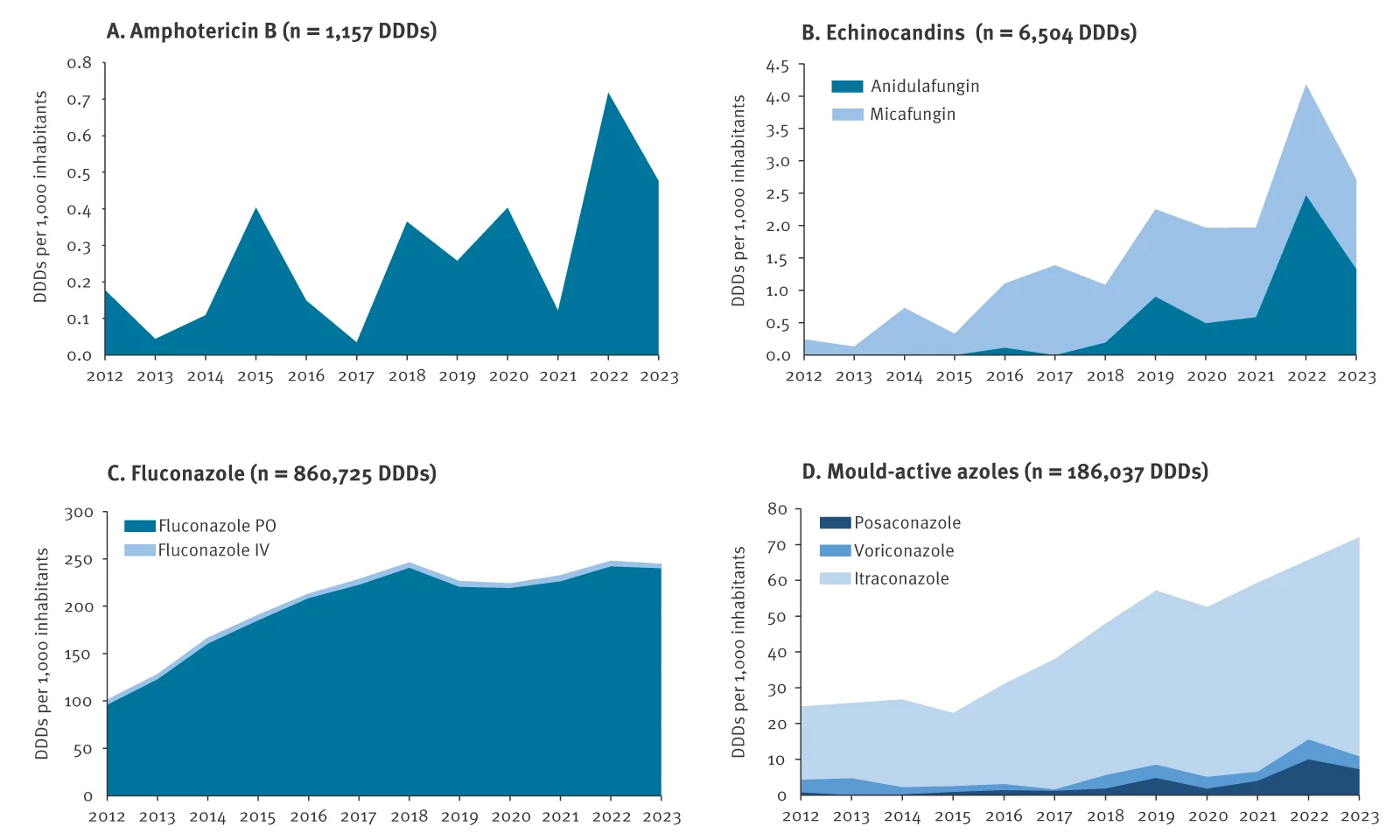
Annual, national use of systemic antifungal compounds, Iceland, 2012–2023

ATC: anatomical therapeutic chemical; DDD: defined daily dose; IV: intravenous administration; PO: oral administration; WHO: World Health Organization.

Panels B–D are stacked graphs so that the vertical thickness of each band represents the use of the corresponding drug or route of administration, whereas the stack's upper boundary shows cumulative use of all drugs together.

The DDDs were defined according to the WHO/ATC definitions as follows: amphotericin B lipid formulations (lipid complex and liposomal formulations) 0.210 g, anidulafungin 0.1 g, micafungin 0.1 g, caspofungin 0.05 g, fluconazole 0.2 g, voriconazole 0.4 g and itraconazole 0.2 g. For posaconazole IV formulation and enterotablets, the WHO/ATC DDD of 0.3 g was used, whereas for posaconazole oral suspension, a corrected DDD of 0.8 g was used, reflecting the daily treatment dose for that formulation. Ketoconazole was not used from 2014 onwards (not depicted). Only lipid formulations of amphotericin B were used in Iceland during the study period. Note that Y-axis scales differ between panels.

The use of systemic azoles increased by 116% during the 12-year study period. Fluconazole use increased from 101 to 246 DDD per 1,000 inhabitants from 2012 to 2018 but remained stable thereafter. The use of mould-active azoles (azoles with activity against moulds; voriconazole, posaconazole and itraconazole) increased from 24.8 to 72.1 DDD per 1,000 inhabitants (191%), explained by an increase in posaconazole and itraconazole use (805% and 200%, respectively).

## Discussion

This population-based, nationwide study provides an important update on trends in the epidemiology of candidaemia in Iceland, both during the current 12-year study period and in a longer-term (2000–2023) perspective. Considering the marked change in age composition of the population over this long period, we performed calculations on ASRs.

Compared with other population-based studies, the average crude incidence rate in Iceland from 2012 to 2023 (5.0 cases/100,000 population) was slightly higher than crude rates reported from most other Northern European countries at analogous time intervals, including Sweden (4.7/100,000; 2015–2016), Norway (3.8/100,000; 2015), Scotland (4.1/100,000; 2012–2013) and England (3.4/100,000; 2015–2024), whereas Denmark has reported higher rates (8.1–8.4/100,000; 2012–2018) [14,19-23]. Nationwide studies in other European countries have reported slightly higher (Greece; 5.6/100,000; 2009–2018) or lower (Switzerland; 4.2/100,000; 2014–2018) incidence rates [24,25]. On the other hand, markedly higher rates have been reported from the US (7.4–8.7/100,000; 2012–2021), whereas Australia has remained a low-incidence country (2.4/100,000; 2014–2015) [5,6,26]. Geographical variations in incidence rates could stem from differences in demographic factors, predisposing conditions, antimicrobial use or healthcare-related factors such as blood culture use and infection control practices.

An important consideration when interpreting long-term trends in candidaemia incidence is the changing age composition of the population. Older individuals are particularly vulnerable to serious *Candida* infections [27], and age standardisation is therefore important in long-term studies to distinguish changes in candidaemia incidence from changes attributable to population age structure. As an example, in Iceland, the proportion of inhabitants older than 60 years increased by almost 40% from 14.3% in 2000 to 19.9% in 2023. Calculations of ASRs for candidaemia in Iceland during that 24-year period, standardised to the Icelandic population in 2023, revealed that although the age-standardised incidence increased significantly from 2012 to 2023 at an average of 7% for each year, the incidence in the first years of that study period, i.e. following the economic crisis in Iceland, was historically low and coincided with a reduction in blood culture frequency and overall severe budget cuts to healthcare. Consequently, the ASR of candidaemia did not change significantly over time when the analysis was extended to the 24-year period and even trended downwards. From a healthcare perspective, however, a stable ASR does not necessarily imply stable case numbers; as the population grows and ages, particularly given the high incidence among older age groups, the absolute number of candidaemia cases and associated healthcare resource needs may increase.

Similar to our findings, rates of invasive candidiasis and candidaemia in the US have generally decreased or plateaued when studied in a long-term perspective (2000–2017), possibly related to improved infection control practices and efforts to prevent catheter-related bloodstream infections [28]. Our findings, however, contrast with results from a meta-analysis showing an increasing overall pooled incidence rate of candidaemia in Europe from 2000 to 2019, although rates were generally lower in northern and western European countries than in southern European countries [4]. More recent increases in candidaemia incidence in some countries may partly reflect factors related to the COVID-19 pandemic and in-hospital transmission of *C. auris*. Severe COVID-19 may have contributed to the increased incidence of candidaemia reported during the pandemic in some countries [22,29], while breaks in infection prevention and control measures are likely to have contributed to the dramatic increase in the number of *C. auris* cases observed in the US and southern Europe in recent years [30,31]. In Iceland, which had one of the lowest mortality rates globally during the pandemic [32], candidaemia incidence also increased during the peak of COVID-19, but detailed analysis of clinical data is needed to ascertain a possible connection. Importantly, the upward trend for incidence coincided with an increased proportion of positive blood cultures, which raises questions about undiagnosed infections taking place at the same time. Therefore, the increase in incidence may still have been somewhat underestimated.

The incidence of candidaemia among < 1-year-old infants decreased substantially during the 24-year period. Likely explanations are the implementation of bundles for central-line care and changes in antifungal prophylaxis practices in the neonatal intensive care unit at the university hospital in the late 2000s. At the other end of the age spectrum, the high incidence rate among very old people, especially males, concurs with reports from many other population studies [5,6,14,22,23]. Potential explanations could be greater exposure to certain risk factors for invasive fungal disease, e.g. invasive surgical procedures, more comorbidities, and less frequent antifungal therapy, whereas the increase between 2020 and 2023 could in part be explained by the COVID-19 pandemic [7,27].

We saw significant sex-related differences in the incidence of candidaemia in Iceland, not only among patients in the oldest age group, but also overall. The ASR among males was significantly higher than among females, both during the current study period and in a 24-year perspective. These findings align with previous studies where adjustments for age and race have been made, and sex differences are increasingly pronounced among patients in older age groups [6,33]. Overall, studies have shown that males are at higher risk of invasive fungal infections [34]. The reasons are unclear but might relate to sex-dependent host immune responses, hormonal factors, or behavioural characteristics affecting risk [35].

Throughout the 12-year study period, *C. albicans* remained the most common species among *Candida* and *Candida*-like isolates cultured from blood in Iceland. It was remarkably low at 41.0%, the lowest reported to date from the Nordic countries and among the lowest reported in population studies in Europe [4,23-25,36]. The significantly reduced proportion of *C. albicans* infections compared with the preceding 12 years was mainly due to an increase in *N. glabratus* (27.2% vs 16.2%) [13]. The proportion of *N. glabratus* was relatively high compared with recent multicentre studies in Europe (2018–2022; 22.3%) and Australia (2010–2019; 21.8%), but closer to surveillance data from England (2020–2024; 27%) and the US (2017–2021; 30.4%) [5,8,33,36]. In our study, the proportion of *N. glabratus* infections did not differ significantly by sex or age group, and the increase in *N. glabratus* can therefore not be explained by the increased incidence of candidaemia among males and patients in the oldest age group. A shift towards other species than *C. albicans*, in particular *N. glabratus*, has been occurring across Europe and in many other parts of the world in the past 25 years, and has been linked to increased use of azoles [8,9,33,36]. As previously reported, fluconazole use in Iceland was 39.5 DDD per 1,000 inhabitants in 2000 [13] and increased more than sixfold in the ensuing 23 years, to 245 DDD per 1,000 inhabitants in 2023, which is probably an important contributor.

In contrast, the proportion of *C. parapsilosis* was relatively low (6.5%) and remained stable, also in a 24-year perspective [13]. Importantly, all *C. parapsilosis* isolates were susceptible to fluconazole. This contrasts with the high proportion of *C. parapsilosis* seen in southern Europe and Latin America and with reports of emerging fluconazole resistance in this species [8-10,24]. Furthermore, no *C. auris* was cultured during the study period. Given the known association of *C. parapsilosis* and *C. auris* with exogenous sources, their low occurrence in Iceland could point to successful infection prevention measures in Icelandic hospitals. Iceland, a relatively isolated country in the mid-Atlantic, has historically experienced delayed importation and emergence of multidrug-resistant microbes, such as MRSA and *C. auris*. However, in February 2026, *C. auris* was isolated from screening samples from a patient transferred to Iceland from an intensive care unit abroad, who subsequently developed a *C. auris* bloodstream infection. This case underscores the importance of ongoing surveillance for emerging multidrug-resistant fungal pathogens in the country.

Overall, 9.3% of isolates were resistant and/or non-WT to fluconazole. Resistance was mainly seen in *N. glabratus* (19%) and *C. tropicalis* (17%), and cross-resistance to other azoles was common. The fluconazole resistance rate among both species was relatively high compared with recent European and US multicentre studies, but the number of isolates in our study was comparatively low [5,8]. Fluconazole resistance has been emerging in clinical *C. tropicalis* isolates, particularly in the Asia-Pacific region, and it is currently classified as a high-priority pathogen by WHO [9,11,26]. The increased proportion of fluconazole-resistant *N. glabratus* isolates in the current study (2012–2023) compared with the preceding 12 years (2000–2011) is concerning, but the isolates from 2000 to 2011 were tested with a gradient diffusion method instead of microbroth dilution, which could potentially have an effect [13].

The proportion of isolates resistant and/or non-WT to echinocandins was low (1.9%) and was 3% in *N. glabratus*. It remains to be determined if these isolates have acquired resistance due to *FKS* gene mutations. The infrequent use of echinocandins during the first half of the study period may have contributed to the low resistance rate. Echinocandin consumption was notably lower in Iceland than in the other Nordic countries, at least until 2019 [23]. A probable reason for the slow start in echinocandin use is the high cost of treatment. From 2018 to 2023, however, the use has increased 2.5-fold. Concurrently, the first multidrug-resistant *Candida* bloodstream isolates have emerged in Iceland.

Limitations of this study include retrospective data collection and missing data on antifungal susceptibility for 1.4% of the bloodstream isolates. Another limitation is that data on antifungal use is based on sales data rather than actual antifungal prescriptions or measurements of antifungal consumption and could not be linked to specific treatment indications. However, the study includes data on all episodes of candidaemia diagnosed in the country, both in university and community hospitals, and data on species is reported for all episodes, allowing us to assess epidemiological and microbiological trends in an unselected group of patients over a long period.

## Conclusion

Despite an apparent increase in incidence in Iceland from 2012 to 2023, candidaemia has remained stable when a long-term perspective is taken and appropriate standardisation is applied for the changing age composition. With an ageing population, the burden of candidaemia has been increasing among the oldest age groups, especially males. Further information on predisposing conditions and healthcare-related risk factors is warranted to elucidate the age and sex disparities seen in this study. Considering the observed shift towards non-*albicans Candida* infections, mainly *N. glabratus*, in the past two decades, and the recent emergence of multidrug-resistant isolates, ongoing epidemiological surveillance is imperative to inform decisions on empirical treatment for candidaemia and to guide prophylaxis strategies and antifungal stewardship practices.

## Data Availability

Aggregated data are presented in the article and supplementary material. National demographic data are publicly available from Statistics Iceland (https://statice.is/statistics/population/inhabitants/overview). Antifungal sales data were obtained from the Icelandic Medicines Agency (https://www.ima.is); requests for additional sales data should be directed to the Agency. Individual-level patient data are not publicly available owing to patient confidentiality. Other data supporting the findings of this study are available from the corresponding author upon reasonable request.

## Supplementary Data

192000 Supplement
