# Supplementary material for: Changes in age composition and epidemiology of candidaemia, Iceland, 2012 to 2023
Source: Euro Surveill. 2026 Oct 8;31(40):2600120. doi: 10.2807/1560-7917.ES.2026.31.40.2600120 (PMC13653211; doi:10.2807/1560-7917.ES.2026.31.40.2600120)
Supplement: Supplementary file 1 [file 26-00120_ASMUNDSDOTTIR_Supplement.pdf]

This supplementary material is hosted by Eurosurveillance as supporting information alongside the article „Changes in age composition and epidemiology of candidaemia, Iceland, 2012 to 2023", on behalf of the authors, who remain responsible for the accuracy and appropriateness of the content. The same standards for ethics, copyright, attributions and permissions as for the article apply. Supplements are not edited by Eurosurveillance and the journal is not responsible for the maintenance of any links or email addresses provided therein.

Data presented here provides additional details on quality control for antifungal susceptibility testing, species distribution and antifungal susceptibility profiles of *Candida* and *Candida*-like bloodstream isolates in Iceland during 2012-2023. Data sources are described fully within the accompanying manuscript.

Supplementary Table S1: Observed minimum inhibitory concentrations (MICs) and acceptable QC ranges for quality control strains used in Sensititre YeastOne microbroth dilution antifungal susceptibility testing.

QC strain	Antifungal agent	Observed MIC(s) (mg/L)[a]	Sensititre YeastOne QC range (mg/L)[b]
Candida parapsilosis ATCC 22019	Amphotericin B	0.25-0.5	0.25-2
	Anidulafungin	0.25-0.5	0.25-2
	Micafungin	0.5	0.5-2
	Fluconazole	1-2	0.5-4
	Voriconazole	0.015-0.03	0.015-0.12
	Posaconazole	0.03-0.12	0.03-0.25
	Itraconazole	0.06-0.25	0.06-0.5
Candida krusei ATCC 6258	Amphotericin B	0.5	0.5-2
	Anidulafungin	0.03-0.12	0.03-0.12
	Micafungin	0.12	0.12-0.5
	Fluconazole	16-32	8-64
	Voriconazole	0.25	0.06-0.5
	Posaconazole	0.25	0.06-0.5
	Itraconazole	0.25	0.12-1

ATCC: American Type Culture Collection; QC, quality control
[a]When all QC measurements yielded the same MIC, a single value is reported rather than a range.
[b]24-hour QC ranges were those specified by the Sensititre YeastOne manufacturer; all matched the Clinical and Laboratory Standards Institute (CLSI) QC ranges except micafungin for *C. krusei* ATCC 6258 (Sensititre YeastOne, 0.12–0.5 mg/L; CLSI M27M44S 4th ed., 0.06–0.25 mg/L).

Supplementary Table S2: Detailed species distribution of *Candida* and *Candida*-like bloodstream isolates in Iceland, 2012-2023 (n = 217).

**Species**	**2012-2015 (n = 44)**		**2016-2019 (n = 71)**		**2020-2023 (n = 102)**		**Total (N = 217)**	
	**n**	**%**	**n**	**%**	**n**	**%**	**n**	**%**
Candida albicans	19	43	33	46	37	36	89	41.0
Nakaseomyces glabratus	12	27	14	20	33	32	59	27.2
Candida tropicalis	6	14	10	14	8	8	24	11.1
Candida dubliniensis	3	7	7	10	10	10	20	9.2
Candida parapsilosis	2	5	4	6	8	8	14	6.5
Clavispora lusitaniae	0	0	2	3	4	4	6	2.8
Meyerozyma guilliermondii	2	5	0	0	1	1	3	1.4
Pichia kudriavzevii	0	0	0	0	1	1	1	0.5
Kluyveromyces marxianus	0	0	1	1	0	0	1	0.5

Because of rounding, percentages may not total 100 in all instances.

Clavispora lusitaniae, *Meyerozyma guilliermondii*, *Pichia kudriavzevii* and *Kluyveromyces marxianus* were formerly known as *Candida lusitaniae*, *Candida guilliermondii*, *Candida krusei* and *Candida kefyr*, respectively.

Supplementary Table S3. Susceptibility of *Candida* and *Candida*-like bloodstream isolates to seven antifungals, Iceland, 2012-2023 (n = 214)

Species and antifungal (no. of isolates tested)	No. of isolates with the given MIC (mg/L)																			R[a]		NWT (if CBP not set)[b]	
	≤0.008	0.015[c]	0.03	0.06	0.12[d]	0.25	0.5	1	2	4	8	>8	16	>16	32	64	128	256	>256	n	%	n	%
***C. albicans* (87)**																							
Amphotericin B	ND				5	46	**36**	0	0	0	0	0	ND							NA	NA	0	0
Anidulafungin	ND	42	33	**11**	1	0	0	0	0	0	0	0	ND							0	0	NA	NA
Micafungin	61	**25**	1	0	0	0	0	0	0	0	0	0	ND							0	0	NA	NA
Fluconazole	ND				3	11	39	**33**	0	0	0	NA	0	NA	1	0	0	0	0	1	1	NA	NA
Voriconazole	40	**44**	1	1	0	0	0	1	0	0	0	0	ND							1	1	NA	NA
Posaconazole	4	14	54	**14**	0	0	1	0	0	0	0	0	ND							NA	NA	1	1
Itraconazole	ND	10	15	45	**16**	0	1	0	0	0	0	NA	0	0	ND					NA	NA	NA	NA
***N. glabratus* (59)**																							
Amphotericin B	ND				2	7	28	**22**	0	0	0	0	ND							NA	NA	0	0
Anidulafungin	ND	13	31	8	**5**	0	1	0	1	0	0	0	ND							2	3	NA	NA
Micafungin	7	**47**	3	1	0	0	0	0	1	0	0	0	ND							1	2	NA	NA
Fluconazole	ND				0	0	0	1	0	4	6	NA	19	NA	18	1	**7**	3	0	11	19	NA	NA
Voriconazole	0	0	1	0	3	13	21	11	**5**	4	1	0	ND							NA	NA	42	71
Posaconazole	0	0	0	1	0	0	4	29	17	0	1	**7**	ND							NA	NA	25	42
Itraconazole	ND	0	0	0	1	3	15	29	4	0	0	NA	1	**6**	ND					NA	NA	7	12
***C. tropicalis* (24)**																							
Amphotericin B	ND				0	0	20	**4**	0	0	0	0	ND							NA	NA	0	0
Anidulafungin	ND	0	4	8	**12**	0	0	0	0	0	0	0	ND							0	0	NA	NA
Micafungin	0	1	**21**	2	0	0	0	0	0	0	0	0	ND							0	0	NA	NA
Fluconazole	ND				0	0	4	10	4	2	1	NA	**1**	NA	1	0	0	0	1	4	17	NA	NA
Voriconazole	0	0	6	9	2	3	**2**	1	0	0	0	1	ND							2	8	NA	NA
Posaconazole	0	0	3	13	2	3	**2**	1	0	0	0	0	ND							NA	NA	6	25
Itraconazole	ND	0	0	9	7	5	**2**	0	1	0	0	NA	0	0	ND					NA	NA	1	4
***C. dubliniensis* (19)**																							
Amphotericin B	ND				6	9	**4**	0	0	0	0	0	ND							NA	NA	0	0
Anidulafungin	ND	6	4	5	**3**	1	0	0	0	0	0	0	ND							NA	NA	1	5
Micafungin	4	8	5	**1**	0	0	1	0	0	0	0	0	ND							NA	NA	1	5
Fluconazole	ND				12	4	**2**	0	0	0	1	NA	0	NA	0	0	0	0	0	NA	NA	1	5
Voriconazole	**18**	0	0	0	0	1	0	0	0	0	0	0	ND							NA	NA	NA	NA
Posaconazole	5	11	1	**1**	0	0	0	1	0	0	0	0	ND							NA	NA	1	5
Itraconazole	ND	12	2	2	**2**	0	1	0	0	0	0	NA	0	0	ND					NA	NA	1	5

Supplementary Table S3 (Continued)

Species and antifungal (no. of isolates tested)	No. of isolates with the given MIC (mg/L)																			R[a]		NWT (if CBP not set)[b]	
	≤0.008	0.015[c]	0.03	0.06	0.12[d]	0.25	0.5	1	2	4	8	>8	16	>16	32	64	128	256	>256	n	%	n	%
C. parapsilosis **(14)**																							
Amphotericin B	ND				3	9	**2**	0	0	0	0	0	ND							NA	NA	0	0
Anidulafungin	ND	0	0	0	0	1	6	**6**	1	0	0	0	ND							0	0	NA	NA
Micafungin	0	0	0	0	0	3	5	**6**	0	0	0	0	ND							0	0	NA	NA
Fluconazole	ND				0	2	4	**7**	1	0	0	NA	0	NA	0	0	0	0	0	0	0	NA	NA
Voriconazole	5	6	**2**	1	0	0	0	0	0	0	0	0	ND							0	0	NA	NA
Posaconazole	1	3	3	4	**3**	0	0	0	0	0	0	0	ND							NA	NA	0	0
Itraconazole	ND	2	5	3	**3**	1	0	0	0	0	0	NA	0	0	ND					NA	NA	0	0
**Other *Candida*/*Candida*-like species (11)[e]**																							
Amphotericin B	ND				5	1	1	**3**	1	0	0	0	ND							NA	NA	0	0
Anidulafungin	ND	0	2	4	0	1	2	**1**	0	0	1	0	ND							0	0	1	9
Micafungin	0	0	3	2	1	3	**1**	0	0	0	1	0	ND							0	0	1	9
Fluconazole	ND				0	0	5	1	0	3	0	NA	0	NA	0	0	0	0	**2**	1	9	2	18
Voriconazole	4	2	1	0	2	0	0	**1**	0	0	0	1	ND							NA	NA	NA	NA
Posaconazole	3	1	1	1	1	2	**1**	1	0	0	0	0	ND							NA	NA	1	9
Itraconazole	ND	3	1	0	2	2	**2**	0	0	0	0	NA	0	1	ND					NA	NA	1	9

CBP: CLSI breakpoint; NA: not applicable; ND: not determined (MIC outside the tested range); R: resistant; WT: wild type; NWT: non-wild type. MIC_{50} underlined, MIC_{90} in bold.
[a] Classification according to CLSI breakpoints (CLSI document M27M44S, 4th ed).
[b] In the absence of established CBP, CLSI epidemiological cutoff value (ECV) was used to distinguish between WT and NWT isolates (CLSI document M57S, 4th ed.).
[c] For anidulafungin and itraconazole the MIC is ≤0.015 mg/L.
[d] For amphotericin B and fluconazole the MIC is ≤0.12 mg/L.
[e] Other *Candida*/*Candida*-like *species* include the indicated number of isolates, as follows: *Clavispora lusitaniae* (*Candida lusitaniae*) n = 6; *Meyerozyma guilliermondii* (*Candida guilliermondii*) n = 3; *Pichia kudriavzevii* (*Candida krusei*) n = 1; *Kluyveromyces marxianus* (*Candida kefyr*) n = 1.
